# Association between inflammatory extension and the ventricular size in adult chronic communicating hydrocephalus: An experimental model of adult hydrocephalus

**DOI:** 10.1186/2045-8118-12-S1-O57

**Published:** 2015-09-18

**Authors:** Ignacio Jusue-Torres, Jennifer Lu, Eric W Sankey, Tito Vivas-Buitrago, Joshua Crawford, Mikhail Pletnikov, Jiadi Xu, Ari Blitz, Barbara Crain, Alicia Hulbert, Hugo Guerrero-Cazares, Oscar Gonzalez-Perez, Alfredo Quiñones-Hinojosa, Pat McAllister, Daniele Rigamonti

**Affiliations:** 1 Johns Hopkins University. School of Medicine, Department of Neurosurgery. USA; 2 Johns Hopkins University, School of Medicine, Department of Psychiatry and Behavioral Sciences, USA; 3 Johns Hopkins University, School of Medicine, F. M. Kirby Research Center for Functional Brain Imaging at the Kennedy Krieger Institute, USA; 4 Johns Hopkins University, School of Medicine, Department of Radiology and Radiological Science, USA; 5 Johns Hopkins University, School of Medicine, Department of Pathology, Division of Neuropathology, USA; 6 Johns Hopkins University, School of Medicine, Department of Oncology, USA; 7 University of Colima , Facultad de Psicologia, Laboratory of Neuroscience, Mexico; 8 Washington University, School of Medicine in St Louis, Department of Neurosurgery, USA

## Introduction

The pathogenesis of normal pressure hydrocephalus (NPH) is not fully understood and the relationship between inflammatory reaction extension and the ventricular enlargement is unknown.

## Methods

Bilateral subarachnoidal injection of kaolin was administered in the cranial convexities of 20 adult rats. MRI was obtained using a Bruker Biospec 11.7 T MRI scanner at 14, 60, 90 and 120 days post kaolin injection. Radiological kaolin extension was defined by the number of kaolin locations showed in the MRI studies. At the end of the experiment, the heads of the rats were decalcified and sliced along with the skull in order to preserve the meninges and bone. A blinded neuropathologist studied the anatomical preparations and analyzed kaolin extension and the inflammatory and fibrotic response.

## Results

Radiological ventricular size showed progressive growth over time at all times (p<0.0001). The fastest ventricular enlargement happened within the first 2 months. Pathological specimens revealed kaolin location at the subarachnoidal space. The extension of the kaolin migration was heterogeneous among rats. Inflammatory and fibrotic response was present at the cranial convexities in all rats, adjacent to superior sagittal sinus in 94% rats, at the interhemispheric fissure in 56% rats, at the Olfactory Bulb in 61% rats, at the anterior basal cisterns in 72% rats, supracerebellar in 56% rats, at the quadrigeminal cisterns in 61% rats, at the lateral midbrain cisterns in 50% rats and within the Virchow-Robin spaces in 61% rats. The extension of the inflammatory response in the subarachnoidal space was associated with ventricular size (p=0.02), and the rate of ventricular enlargement (p=0.03).

## Conclusions

The extension of the inflammatory response to kaolin injected in the subarachnoidal space is associated with ventricular size and the rate of ventricular enlargement in adult rats.
